# ADVERSE CHILDHOOD EXPERIENCES AND DEMENTIA AMONG OLDER US ADULTS: ASSESSMENT OF GENDER AND RACIAL DISPARITIES

**DOI:** 10.1093/geroni/igae098.0787

**Published:** 2024-12-31

**Authors:** Monique Brown

**Affiliations:** University of South Carolina, Columbia, South Carolina, United States

## Abstract

Six in ten US adults report exposure to adverse childhood experiences (ACEs). However, their effects on health outcomes are seen across the life course. ACEs have been linked to cognitive impairment. However, less is known about the association between ACEs and a dementia diagnosis. In addition, gender and racial disparities may exist in this relationship. Therefore, this study aimed to determine the association between ACEs and dementia, and to assess the related gender and racial disparities. Data were obtained from the 2012-2020 Health and Retirement Study (N=4,235 respondents aged 51 and older). ACEs assessed included emotional neglect, parental substance abuse, physical abuse, economic adversity, academic challenges, and problems with the law. Dementia was operationalized as ever having dementia. Crude and adjusted logistic regression models were used to determine the associations between ACEs and dementia. Additive and multiplicative interaction effects by gender and race were tested. Approximately 70% of participants reported ACEs and 3% reported ever having dementia. After adjusting for sociodemographic factors, depression, and gender and race interactions, individuals who reported ACEs had 15 times higher odds (aOR: 14.5; 95%CI: 1.56 – 133.9) of ever having dementia compared to individuals without ACEs. Interaction by gender was statistically significant where the relationship between ACEs and dementia was statistically significant for men (aOR: 3.29; 95%CI: 1.28 – 8.42) but not for women. Interaction by race was not statistically significant. Trauma-informed interventions addressing ACEs may reduce the odds of dementia onset, especially for men. Future research should examine pathways between ACEs and dementia.

